# Sintilimab plus chemotherapy with or without bevacizumab biosimilar IBI305 in EGFR-mutated non-squamous NSCLC patients who progressed on EGFR TKI therapy: A China-based cost-effectiveness analysis

**DOI:** 10.1371/journal.pone.0312133

**Published:** 2024-10-18

**Authors:** Juan Peng, Huiling Xu, Qiao Liu

**Affiliations:** 1 Department of Pharmacy, Jiangxi Provincial People’s Hospital, The First Affiliated Hospital of Nanchang Medical College, Nanchang, China; 2 Department of Pharmacy, The Second Xiangya Hospital of Central South University, Changsha, China; The University of Texas MD Anderson Cancer Center, UNITED STATES OF AMERICA

## Abstract

**Background:**

This study aims to compare the cost-effectiveness of sintilimab in combination with chemotherapy, with or without bevacizumab biosimilar IBI305, versus chemotherapy alone for patients with epidermal growth factor receptor (EGFR)-mutated non-small-cell lung cancer (NSCLC) who have progressed on tyrosine-kinase inhibitor (TKI) treatment from the perspective of the Chinese healthcare system.

**Methods:**

10-year Markov model was developed using a 21-day cycle length. Transition probabilities were derived from the ORIENT-31 trial, while cost and health state utilities were obtained from publicly databases, local hospitals, and published literature. Incremental cost-effectiveness ratios (ICERs) were calculated as the primary model output and compared to a willingness-to-pay (WTP) threshold range of $15,289.34 to $38,223.34 per quality-adjusted life-years (QALY). Sensitivity analyses were performed to assess the robustness of the model.

**Results:**

In the base-case analysis, sintilimab plus IBI305 and chemotherapy had an ICER of $53,266.32/QALYs, exceeding the upper WTP threshold. Sintilimab plus chemotherapy had an ICER of $15,329.11/QALY, slightly above the lower WTP threshold. Subgroup analysis yielded consistent results. Deterministic sensitivity analyses found no ICER for sintilimab plus chemotherapy beyond the upper WTP threshold. Most model input changes did not decrease the ICER of sintilimab plus IBI305 and chemotherapy below the upper WTP threshold. Probabilistic sensitivity analyses further demonstrated the cost-effectiveness superiority of sintilimab plus chemotherapy over sintilimab plus IBI305 and chemotherapy.

**Conclusion:**

This study supports the cost-effectiveness of using sintilimab in combination with chemotherapy. Nevertheless, the cost-effectiveness of combining sintilimab with IBI305 and chemotherapy in this particular patient group may be lacking.

## Introduction

Epidermal growth factor receptor (EGFR) mutations are prevalent in approximately half of Asian patients diagnosed with lung adenocarcinoma [1,2]. In the case of EGFR-mutated non-small-cell lung cancer (NSCLC), the standard first-line treatment involves EGFR-targeted therapy using tyrosine-kinase inhibitors (TKI). However, the development of drug resistance and subsequent disease progression is inevitable [3]. Limited treatment options exist for patients who have progressed after receiving third-generation EGFR TKI, or those without Thr790Met mutations progressing after treatment with first-generation or second-generation EGFR TKIs [4]. In China, platinum-based doublet chemotherapy with or without bevacizumab is the standard-of-care for these patients [5], but it offers minimal survival advantages. Consequently, there is an urgent need for more efficacious therapeutic strategies designed for EGFR-mutated NSCLC patients exhibiting resistance to TKI treatment.

The first interim analysis of the ORIENT-31 study has revealed promising results regarding the efficacy and tolerability of a combination therapy involving the anti-PD-1 antibody sintilimab, VEGF inhibitor bevacizumab biosimilar IBI305, and chemotherapy in this patient population [6]. Subsequent analysis from the same study demonstrated that sintilimab plus chemotherapy also exhibited significant and clinically meaningful survival advantages while maintaining an optimal safety profile [7]. However, uncertainty remains regarding the widespread clinical application of these two promising therapeutic strategies due to the potential economic burden imposed by the expensive anti-PD-1 antibody sintilimab. Therefore, conducting a cost-effectiveness analysis is necessary to determine whether these new therapies offer survival benefits at an affordable cost.

This study aims to compare cost and effectiveness of sintilimab plus chemotherapy, with or without bevacizumab biosimilar IBI305, against chemotherapy alone for in patients with EGFR-mutated NSCLC who progressed on EGFR TKI treatment from the Chinese healthcare perspective. The research findings will provide useful evidence to inform clinical decision-making and optimize treatment strategies for EGFR-mutated NSCLC patients resistant to TKI treatment.

## Material and methods

### Overview

This economic evaluation utilized a Markov model to estimate the cost-effectiveness of three treatment strategies for patients with EGFR-mutated NSCLC who had progressed on EGFR TKI treatment. The treatment options considered were sintilimab plus IBI305 and chemotherapy, sintilimab plus chemotherapy, and chemotherapy alone. Chemotherapy was included as the comparator, as it is the recommended treatment option for this patient population according to the Chinese Society of Clinical Oncology (CSCO) [5].

To ensure methodological rigor, this study adhered to the China Guidelines for Pharmacoeconomic Evaluations (2020 Edition) and followed the Consolidated Health Economic Evaluation Reporting Standards (CHEERS) reporting guideline for the design, analysis, and reporting of our model [8,9].As no identifiable data was used and there was no direct interaction or intervention with human subjects, the study was exempt from ethical review by the Clinical Ethics Committee of the Jiangxi Provincial People’s Hospital (The First Affiliated Hospital of Nanchang Medical College) according to the Measures for Ethical Review of Life Science and Medical Research Involving Humans (2023). The study was conducted in accordance with the Declaration of Helsinki (as revised in 2013).

The perspective of the Chinese healthcare system was adopted for this study. The model assessed the total cost of treatment per patient, the effectiveness measured in quality-adjusted life-years (QALYs), and the incremental cost-effectiveness ratio (ICER) between the two sintilimab-containing treatment strategy compared to chemotherapy alone. Both costs and effectiveness outcomes were discounted at an annual rate of 5% [8].

### Markov model

**Fig 1:**
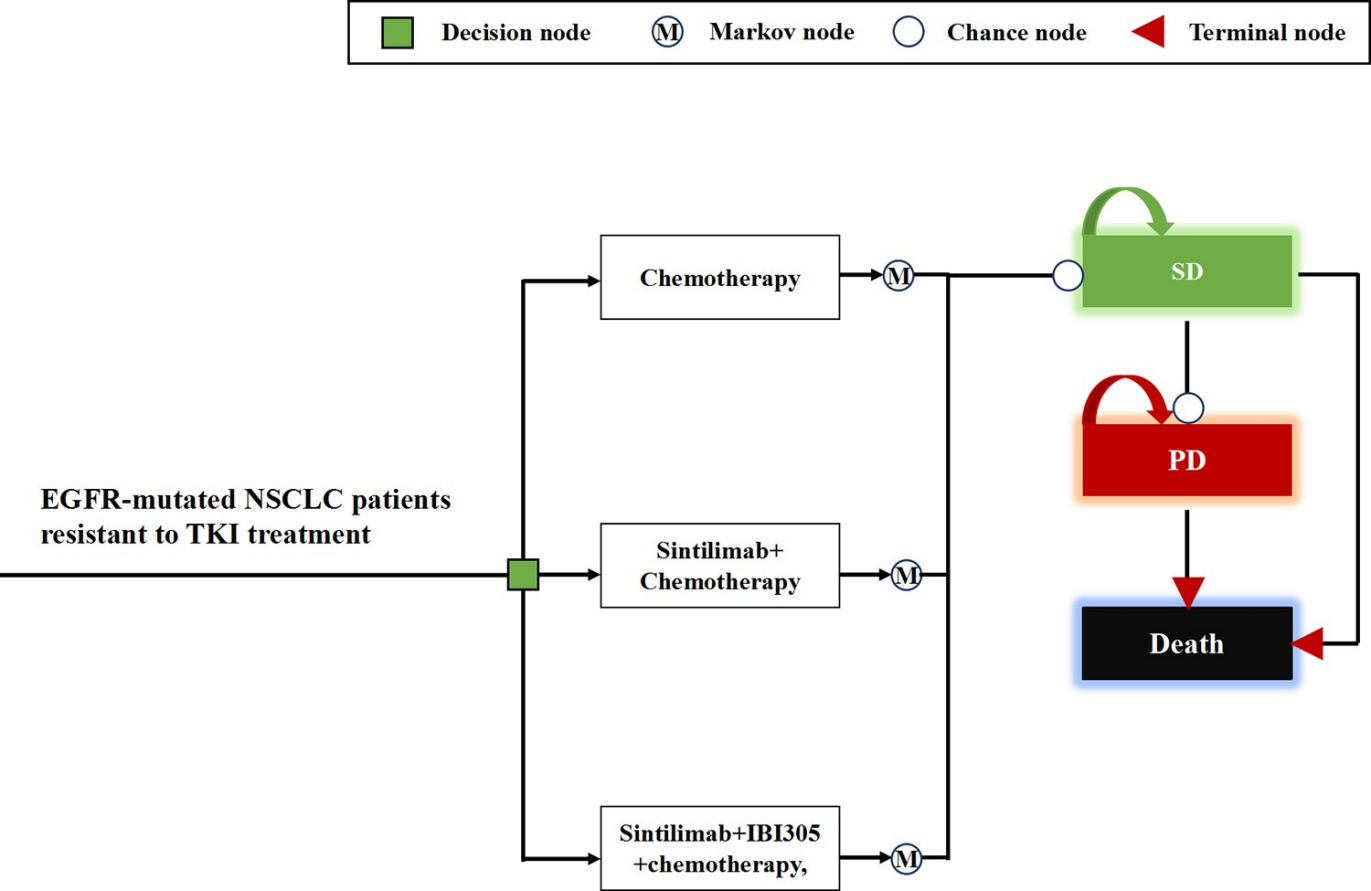
Diagram of Markov model. EGFR, epidermal growth factor receptor; NSCLC, non-small-cell lung cancer; TKI, tyrosine-kinase inhibitor; SD, stable disease; PD, progressed disease.

The Markov model used in the study simulated the progression of patients through three mutually exclusive health states: stable disease (SD), progressed disease (PD), and death, as depicted in **Fig 1**. Initially, all patients started in the SD health state and were randomly assigned to one of the following treatment options: sntilimab plus IBI305 and chemotherapy; sintilimab plus chemotherapy; or chemotherapy alone. Specific dosage and administration schedules for each treatment option can be found in **S1 Table**.

Within each Markov cycle, which lasted 21 days to align with the treatment administration interval specified in the ORIENT-31 trial [6,7], patients had the possibility of transitioning to the PD health state due to disease progression or facing mortality. In the PD health state, patients could receive sub-anticancer therapies following the CSCO guidelines [5]. Moreover, all patients received best supportive care (BSC), and palliative care was considered prior to imminent death.

The time horizon of the Markov model spanned 10 years, chosen based on the median overall survival (OS) observed in the ORIENT-31 trial. The trial reported OS of approximately 20 months for the three treatment strategies compared in the model: sintilimab plus IBI305 and chemotherapy, sintilimab plus chemotherapy, and chemotherapy alone [7].

### Transition probability

In the chemotherapy arm of the ORIENT-31 study, we estimated the transition probabilities between three health states using the Kaplan-Meier (KM) survival curves. To accomplish this, we first digitized the progression-free survival (PFS) and OS data from the KM curves, which allowed us to generate pseudo-individual patient data [10]. We then performed a series of goodness-of-fit tests, including evaluating the Akaike information criterion and Bayesian information criterion, as well as visually assessing modeled curves against KM curves. These tests helped us identify the optimal parametric survival distribution among five commonly used distributions (exponential, Weibull, log-normal, log-logistic, and Gompertz) for the reconstructed data. The results of these goodness-of-fit tests can be found in **S2 Table and S1 and S2 Figs**. After selecting the log-logistic distribution to fit and extrapolate the survival of the chemotherapy arm, we used the parameters theta (θ) and kappa (κ) to derive the transition probabilities between health states. For a given time cycle t, the survival probabilities were calculated as follows: $S(t)=1/[1+\exp(\theta )t^{\kappa }]$.

For the sntilimab plus IBI305 and chemotherapy; sintilimab plus chemotherapy arms, we obtained the hazard ratios (HRs) for their PFS and OS compared to chemotherapy alone. To adjust their survival probabilities, we used a specific formula: $S(t)=S{{\left(t\right)}_{\mathrm{reference}}}^{HR}$ [11]. Similarly, the HRs estimated for different subgroups based on age, sex, baseline Eastern Cooperative Oncology Group (ECOG) performance status scores, baseline brain metastasis status, Thr790Met mutation status, smoking status, baseline liver metastases status, previous EGFR tyrosine-kinase inhibitor treatment status, and EGFR mutation status were used to estimate subgroup-level transition probabilities. Model inputs related to the estimation of transition probabilities can be found in **S3 Table**.

### Costs

In this study, cost estimation for cancer treatment considered various factors, including drug acquisition, adverse events (AEs) management, sub-anticancer therapies, routine follow-up, BSC and palliative care. We reported medical costs for each strategy in 2022 US dollars.

To calculate the drug acquisition costs per cycle, we retrieved the latest bid-winning prices for specific drugs from the National Health Industry Data Platform [12]. The costs were determined based on the dosages administration per treatment cycle, as listed in **S1 Table**. For drugs with dosages dependent on anthropometry (e.g., weight or body surface area), the targeted patients were modeled to have an average weight of 69.6 kg for males and 59.0 kg for females [13], along with an average body surface area of 1.72 m^2^ [14]. The medical costs associated with managing of grade≥3 AEs were estimated through several steps: Firstly, the charging items for treating each AEs were identified based on either Chinese expert consensus or local oncologists’ opinions [15–22]. Relevant costs were obtained from local general hospitals **(S4 Table**). Then, the AEs management cost for each treatment arm was estimated by multiplying the frequency of grades 3/4 AEs by the estimated cost for each AE. The costs related to sub-anticancer therapies, routine follow-up, BSC and palliative care were obtained from published literature [14].

### Health state utilities

Chinese-specific health utilities were used in the model, with a score of 0.70300 assigned to the PD heath state [23]. The score for the SD health state for each treatment arm varied depending on the patient’s previous EGFR tyrosine-kinase inhibitor treatment status (i.e., the number of lines of TKI treatment received before).

To evaluate the negative effects of grades 3/4 AEs resulting from each treatment strategy on health state utilities, a frequency-weighted sum approach was employed. The disutility for each AE was obtained from the Institute for clinical and economic review [24], while the duration for each AE was derived from published papers (**S5 Table**) [25–29]. The same algorithm used for estimating AE management costs was utilized for calculating the impact of grades 3/4 AEs on health state utilities.

The calculation of grades 3/4 AEs-induced costs and utilities for each treatment arm is provided in **S6 Table**. All model inputs related to costs and health state utilities estimation were summarized in **S7 Table**.

### Statistical analysis

#### Base-case ICERs

The statistical tools for this cost-effectiveness analysis involved using treeAge Pro Healthcare software (version 2022, https://www.treeage.com/) and R software (version 4.0.4, http://www.r-project.org). The study aimed to determine the relative cost-effectiveness of sntilimab plus IBI305 and chemotherapy compared to chemotherapy, as well as sintilimab plus chemotherapy versus chemotherapy alone. This comparison was done by calculating the ICERs and comparing them to a predetermined willing-to-pay (WTP) threshold.

Since there was no explicit WTP threshold benchmark established for ICER-based decisions in China, the study followed the recommendations provided by Cai et al [30]. A range of 1.2 to 3.0 times China’s per capita gross domestic product (GDP) in 2022 was used as the potential WTP threshold, corresponding to a value of $15,289.34 to $38,223.34 per QALY [31]. A strategy with an ICER below the predetermined WTP threshold was considered cost-effective, while a strategy with an ICER above the threshold was considered non-cost-effective.

#### Subgroup-level ICERs

Subgroup-level ICERs were also analyzed to explore the cost-effectiveness results for the two sintilimab-containing treatment strategies at a subgroup level. The subgroup-level hazard ratios (HRs) of PFS for these treatment arms, which have been detailed in **S3 Table**, were used in these analyses.

#### Sensitivity analysis

To assess the robustness of the cost-effectiveness results, two sensitivity analyses were performed. In the deterministic sensitivity analyses (DSA) performed, the impact of uncertainty associated with individual model inputs was assessed by varying their value within plausible ranges. This included using 95% confidence intervals for HRs, 0% to 8% for discount rate and ±25% for other model inputs. Probabilistic sensitivity analyses (PSA) were also conducted using 10,000 Monte Carlo simulations to investigate the influence of the uncertainties in multiple model inputs on the findings. Parameter distributions for model inputs in the PSA were determined as proposed by the International Society for Pharmacoeconomics and outcomes Research and the Society for Medical Decision Making Modeling Good Research Practices Task Force [32]. The ranges for DSA and distributions for PSA were outlined in **S3 and S7 Tables**.

## Results

### Base-case ICERs

In patients with EGFR-mutated NSCLC who had progressed on EGFR TKI treatment, both sntilimab plus IBI305 and chemotherapy, as well as sintilimab plus chemotherapy, demonstrated increased costs and improved survival compared to chemotherapy alone (**Table 1**). The cost and survival improvements for sntilimab plus IBI305 and chemotherapy were $20,713.72 and 0.38887 QALYs, while for sintilimab plus chemotherapy, the improvements were $5,964.73 and 0.38911 QALYs, respectively.

**Table 1:** Base-case analysis results.

Strategy	Chemotherapy	Sintilimab+chemotherapy	Sintilimab+IBI305+chemotherapy
**Costs**	21,322.48	27,287.22	42,036.20
**LYs**	2.29485	2.82935	2.80058
**QALYs**	1.64024	2.02936	2.02912
**Incremental costs (vs chemotherapy)**	NA	5,964.73	20,713.72
**Incremental LYs (vs chemotherapy)**	NA	0.53450	0.50573
**Incremental QALYs (vs chemotherapy)**	NA	0.38911	0.38887
**ICER (LYs per QALY)**	NA	51,052.10	83,120.37
**ICER (QALYs per QALY)**	NA	15,329.11	53,266.32

Abbreviations:LYs: Life years QALYs, quality-adjusted life-years; ICERs, incremental cost-effectiveness ratios; NA, not applicable.

The ICER for sntilimab plus IBI305 and chemotherapy was $53,266.32/QALYs, surpassing the upper bound of the preset WTP threshold range ($38,223.34 per QALY). For sintilimab plus chemotherapy, the ICER was $15,329.11/QALY, slightly higher than the lower bounds of the preset WTP threshold range ($15,289.34 per QALY) (**Table 1**).

### Subgroup-level ICERs

As shown in **S8 Table,** both sintilimab-containing treatment strategies resulted in significant increases in costs and survival outcomes across all subgroups. The ICERs for sntilimab plus IBI305 and chemotherapy ranged from $44,635.66 to $58,128.45/QALY, consistently exceeding the upper bounds of the preset WTP threshold range. Conversely, the ICERs for sntilimab plus chemotherapy ranged from $13,198.49 ~$16,351.05 /QALY, aligning with or nearing the lower bounds of the preset WTP threshold range.

### Sensitivity analysis

**Fig 2:**
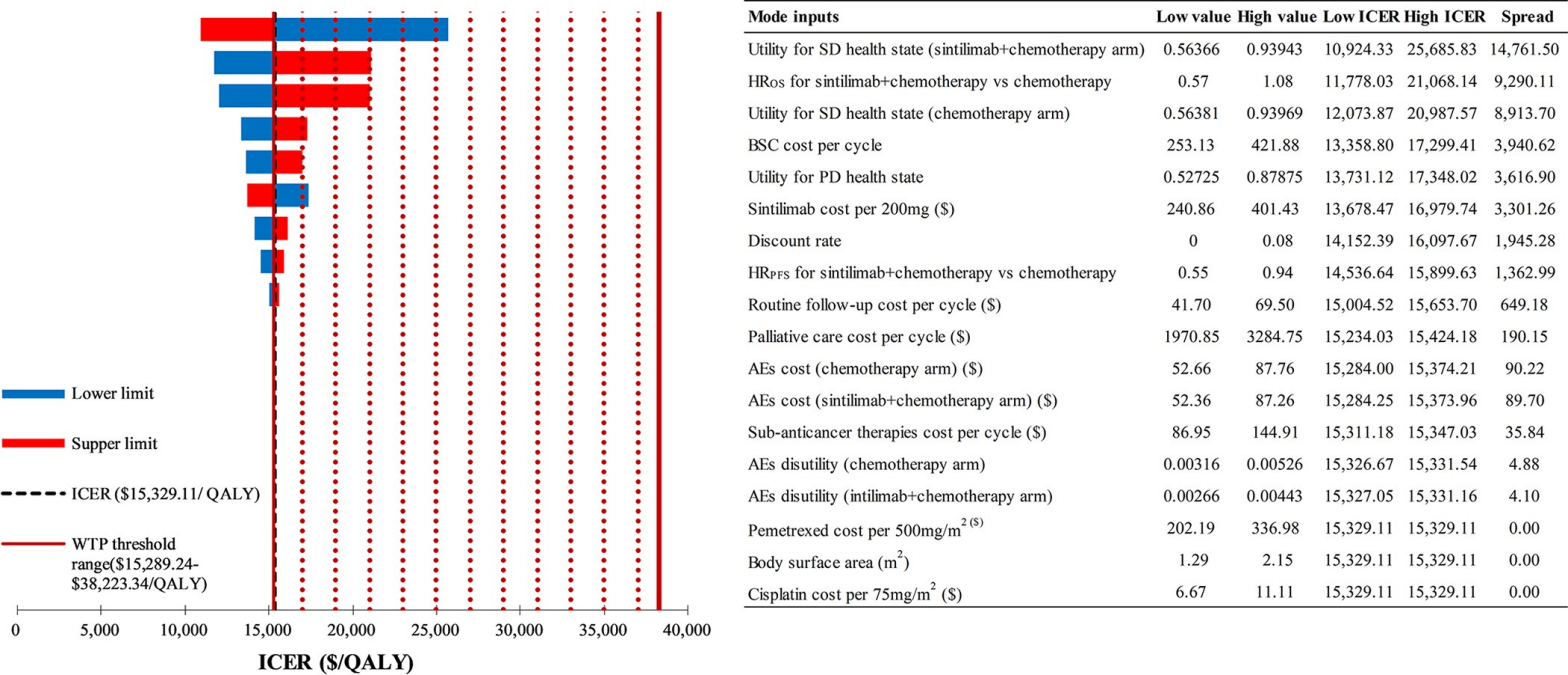
DSA results for sintilimab+chemotherapy vs chemotherapy alone. DSA, deterministic sensitivity analysis; ICER, incremental cost-effectiveness ratio; WTP, willingness-to-pay; QALY, quality-adjusted life-year; SD, stable disease; HR, hazard ratio; OS, overall survival; BSC, best supportive care; PD, progressed disease; PFS, progression-free survival; AEs, adverse events.

**Fig 3:**
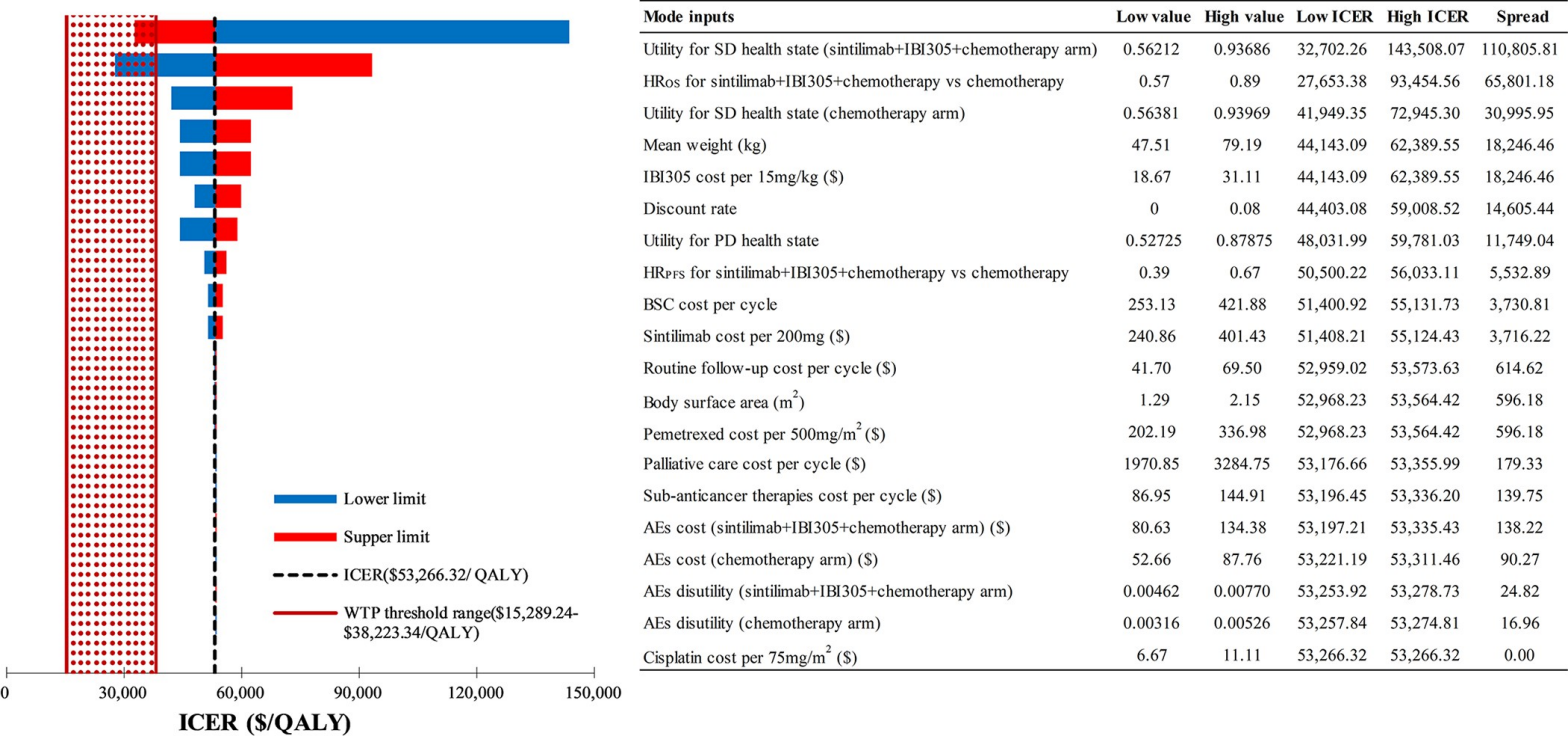
DSA results for sintilimab+IBI305+chemotherapy vs chemotherapy alone. DSA, deterministic sensitivity analysis; ICER, incremental cost-effectiveness ratio; WTP, willingness-to-pay; QALY, quality-adjusted life-year; SD, stable disease; HR, hazard ratio; OS, overall survival; BSC, best supportive care; PD, progressed disease; PFS, progression-free survival; AEs, adverse events.

The DSA results from **Figs 2 and 3** emphasize the significant impact of utility for SD health state and OS HR on the ICERs of sintilimab-containing treatment strategies compared to chemotherapy alone. Notably, substantial changes in all model inputs did not result in the ICER between sintilimab plus chemotherapy and chemotherapy exceeding the upper bound of the preset WTP threshold range ($38,223.34 per QALY). Furthermore, with the exception of the utility for SD health state of the sintilimab+IBI305+chemotherapy arm and OS HR, other changes in model inputs did not decrease the ICER between sintilimab plus IBI305 and chemotherapy below the upper bound of the preset WTP threshold range.

**Fig 4:**
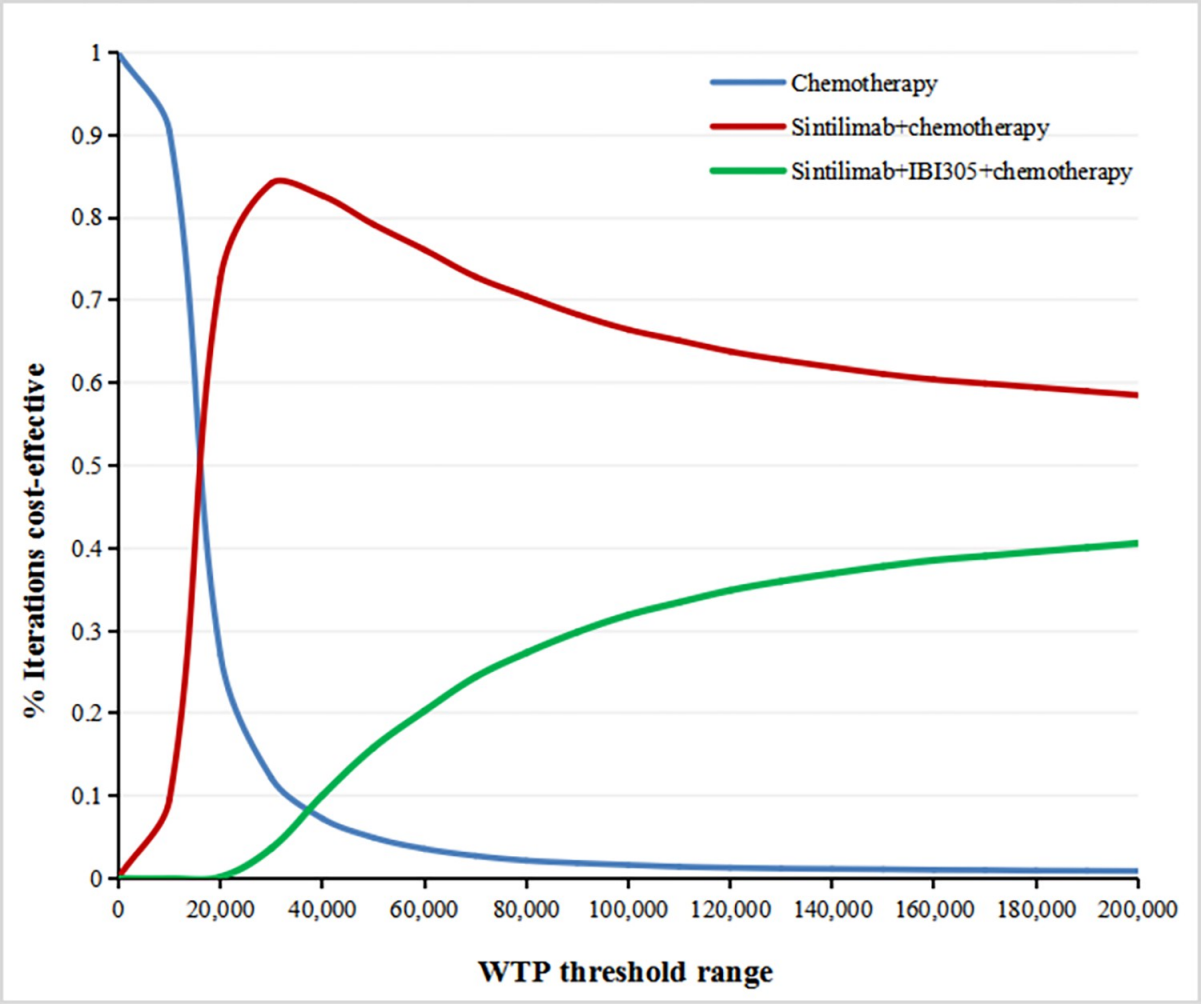
PSA results for total patients with EGFR-mutated NSCLC who had progressed on EGFR TKI treatment. PSA, probabilistic sensitivity analyses; EGFR, epidermal growth factor receptor; NSCLC, non-small-cell lung cancer; TKI, tyrosine-kinase inhibitor.

The PSA results were visualized using cost-effectiveness acceptability curves, as shown in **Fig 4**. Our analysis revealed that the probability of sintilimab plus chemotherapy being cost-effective exhibits an initial significant increase followed by a subsequent decrease. Importantly, throughout this WTP threshold range of $0 to $200,000 per QALY, the probability of sintilimab plus chemotherapy being cost-effectiveness consistently remains higher than the probability of sintilimab plus IBI305 and chemotherapy being cost-effectiveness as the WTP threshold increases.

## Discussion

### Principal findings

This research aimed to systematically compare the cost-effectiveness of two treatment strategies: sintilimab plus IBI305 and chemotherapy, and sintilimab plus chemotherapy versus chemotherapy alone, in Chinese patients with EGFR-mutated NSCLC who had progressed on EGFR TKI treatment. The study sought to provide valuable insights into the optimal therapy for this patient population. The key findings are as follows:

- Base-case analysis demonstrated that both sintilimab-containing treatment strategies resulted in increased costs and improved survival compared to chemotherapy alone. However, the incremental cost-effectiveness ratio (ICER) for sintilimab plus IBI305 and chemotherapy exceeded the upper bounds of the willingness-to-pay (WTP) threshold range ($38,223.34 per QALY), suggesting it may not be considered cost-effective. In contrast, the ICER for sintilimab plus chemotherapy fell within the WTP threshold range ($15,289.34 to $38,223.34 per QALY), indicating it may be a cost-effective option. This finding supports the use of sintilimab plus chemotherapy as the preferred treatment strategy for this patient population.
- Subgroup-level analysis consistently showed that the ICERs for sintilimab plus IBI305 and chemotherapy ($44,635.66 to $58,128.45 per QALY) exceeded the upper bounds of the preset WTP threshold range across all subgroups. This suggests that sintilimab plus IBI305 and chemotherapy may not be cost-effective in all patient subgroups. Conversely, the ICERs for sintilimab plus chemotherapy ($13,198.49 to $16,351.05 per QALY) consistently aligned with or approached the lower bounds of the WTP threshold range, indicating it was cost-effective regardless of subgroups. These findings provide stronger evidence supporting the use of sintilimab plus chemotherapy in different patient subgroups.
- DSA results revealed that even with substantial changes to all model inputs, the ICER for sintilimab plus chemotherapy did not exceed the upper bound of the preset WTP threshold range. Similarly, when considering the comparison between sintilimab plus IBI305 and chemotherapy alone, the ICER did not fall below the upper bound of the WTP threshold range for most changes in model inputs, except for the utility for SD health state of the sintilimab+IBI305+chemotherapy arm and OS HR. These findings supported our base-case and subgroup-level analysis results.
- The PSA results further highlight the superior cost-effectiveness of sintilimab combined with chemotherapy over sintilimab plus IBI305 and chemotherapy, showing a higher probability of cost-effectiveness for the former across the WTP threshold range of $0 to $200,000 per QALY. This advantage likely arises from the significantly lower cost of sintilimab plus chemotherapy ($27,287.22 vs. $42,036.20) and its marginally better efficacy (2.02936 QALY vs. 2.02912 QALY), leading to sintilimab plus chemotherapy dominating the sintilimab plus IBI305 and chemotherapy regimen.

### Strengths and limitation

This study has several notable strengths that contribute to its significance. ***First*,** this research is the first economic evaluation to compare the cost and clinical outcomes of sintilimab plus IBI305 and chemotherapy, and sintilimab plus chemotherapy versus chemotherapy alone in Chinese patients with EGFR-mutated NSCLC who have progressed on EGFR TKI treatment. The study fills a gap in the literature by providing valuable insights into the relative cost-effectiveness of these treatment strategies. ***Second*,** the evaluation of cost-effectiveness across different subgroups is a notable strength of this manuscript. By analyzing subgroups, the study provides a deeper understanding of how these treatments may impact different populations or specific demographic characteristics. This information is crucial for decision-makers, policymakers, and healthcare providers when making informed decisions about resource allocation and treatment selection. ***Third*,** the study systematically considers the impact of AEs in the model. It takes into account the negative consequences of AEs on both health state utilities and additional treatment costs. This comprehensive evaluation of AEs enhances the accuracy and reliability of the cost-effectiveness analysis. ***Fourth***, by incorporating local expert-recommended treatment items and locally derived costs, this study provides valuable insights into the economic impact of AEs within the Chinese healthcare context. This localized approach enhances the relevance and applicability of the findings for decision-making in the Chinese healthcare system.

This study also has several limitations. ***First***, the study relied on health state utilities derived from existing literature. The DSA conducted demonstrated that variations in health utilities within a reasonable range can have a significant impact on the ICERs. However, it is noted that these variations may not change the conclusions of the model. Nonetheless, obtaining more accurate data on health utilities is important to improve the precision of the study’s results. ***Second***, our study was conducted based on the ORIENT-31 trial, which exclusively compared sintilimab plus IBI305 and chemotherapy, sintilimab plus chemotherapy, and chemotherapy alone. Consequently, this economic evaluation did not incorporate other treatment strategies, such as bevacizumab plus chemotherapy, despite its status as the standard-of-care for patients with EGFR-mutated NSCLC who have progressed on EGFR TKI treatment. ***Third*,** a limitation of our study is that model costs were projected for 10 years without accounting for potential discontinuations or the future introduction of lower-cost biosimilars. Nonetheless, our sensitivity analysis demonstrated that even with significant changes to all model cost inputs, the ICER for sintilimab plus chemotherapy versus chemotherapy remained below the upper limit of the predefined WTP threshold range. This indicates that our findings are robust despite this limitation. ***Fourth*,** the uniqueness of the Chinese health system and economic environment may limit the generalizability of the study’s findings to other contexts. It is crucial to recognize this limitation when considering the applicability of the study’s results to different populations or healthcare systems. Nevertheless, given that China represents a substantial proportion of the world’s lung cancer patients, the findings of this study remain relevant in addressing the global burden associated with this disease.

## Conclusion

From the Chinese healthcare perspective, this study supports the use of sintilimab plus chemotherapy as a cost-effective treatment strategy for patients with EGFR-mutated NSCLC who have progressed on EGFR TKI treatment. The findings also highlight the potential lack of cost-effectiveness for sintilimab plus IBI305 and chemotherapy in this specific patients population.

## Supporting information

S1 Table Treatment strategies compared in the model. (DOCX)

S2 Table AIC and BIC statistics for survival fitting of the chemotherapy arm. (DOCX)

S3 Table Model inputs regarding transition probabilities estimation. (DOCX)

S4 Table Derivation of AEs-related management costs. (DOCX)

S5 Table Derivation of AEs-related utility decrements. (DOCX)

S6 Table The calculation of grades 3/4 AEs-induced costs and utilities for each treatment arm. (DOCX)

S7 Table Model inputs regarding costs and health state utilities estimation. (DOCX)

S8 Table Subgroup-level analysis results. (DOCX)

S1 Fig KM curve and modeled curves for the OS of the chemotherapy arm. KM, Kaplan-Meier; OS, overall survival.
(TIF)

S2 Fig KM curves and modeled curves for the PFS of the chemotherapy arm. KM, Kaplan-Meier; PFS, progression-free survival.
(TIF)
