# Supplementary material for: Sintilimab plus chemotherapy with or without bevacizumab biosimilar IBI305 in EGFR-mutated non-squamous NSCLC patients who progressed on EGFR TKI therapy: A China-based cost-effectiveness analysis
Source: PLoS One. 2024 Oct 18;19(10):e0312133. doi: 10.1371/journal.pone.0312133 (PMC11488704; doi:10.1371/journal.pone.0312133)
Supplement: S1 Table — (DOCX) [file pone.0312133.s001.docx]

**S1 Table. Treatment strategies compared in the model**

| **Treatment** | **Dosage** | **Administration schedule** |
| --- | --- | --- |
| Chemotherapy | Pemetrexed, 500 mg/m² | On day 1 of each 3-week cycle |
|  | Cisplatin, 75 mg/m² | On day 1 of each 3-week cycle for four cycles |
| Sintilimab+chemotherapy | Sintilimab, 200mg | On day 1 of each 3-week cycle |
|  | Chemotherapy is the same as above | |
| Sintilimab+IBI305+ chemotherapy | Sintilimab, 200mg | On day 1 of each 3-week cycle |
|  | IBI305, 15mg/kg | On day 1 of each 3-week cycle |
|  | Chemotherapy is the same as above | |
