# Supplementary material for: Sintilimab plus chemotherapy with or without bevacizumab biosimilar IBI305 in EGFR-mutated non-squamous NSCLC patients who progressed on EGFR TKI therapy: A China-based cost-effectiveness analysis
Source: PLoS One. 2024 Oct 18;19(10):e0312133. doi: 10.1371/journal.pone.0312133 (PMC11488704; doi:10.1371/journal.pone.0312133)
Supplement: S2 Table — (DOCX) [file pone.0312133.s002.docx]

**S2 Table. AIC and BIC statistics for survival fitting of the chemotherapy arm**

| **Parametric survival distribution** | **OS data** | | **PFS data** | |
| --- | --- | --- | --- | --- |
|  | **AIC** | **BIC** | **AIC** | **BIC** |
| Exponential | -303 | -298 | -156 | -156 |
| Weibull | -370 | -363 | -182 | -182 |
| Log-normal | -404 | -397 | -216 | -216 |
| Log-logistic^a^ | **-421** | **-414** | **-231** | **-231** |
| Gompertz | -388 | -378 | -225 | -225 |

Abbreviations: OS, overall survival; PFS, progression-free survival; AIC, Akaike information criterion; BIC, Bayesian information criterion.

^a^The log-logistic distribution was associated with the lowest AIC and BIC values, therefore it is considered to provide the best fit for first-line chemotherapy.
