# Supplementary material for: Sintilimab plus chemotherapy with or without bevacizumab biosimilar IBI305 in EGFR-mutated non-squamous NSCLC patients who progressed on EGFR TKI therapy: A China-based cost-effectiveness analysis
Source: PLoS One. 2024 Oct 18;19(10):e0312133. doi: 10.1371/journal.pone.0312133 (PMC11488704; doi:10.1371/journal.pone.0312133)
Supplement: S3 Table — (DOCX) [file pone.0312133.s003.docx]

**S3 Table. Model inputs regarding transition probabilities estimation**

| **Model inputs** | **Baseline Value** | **Range** | **Distribution** | **Source** |
| --- | --- | --- | --- | --- |
| **Survival parameters** | | | | |
| OS of the chemotherapy arm | Log-logistic θ=0.005473;κ=1.567700 | Fixed in DSA | Fixed in PSA | Parametric survival analyses of ORIENT-31 data |
| PFS of the chemotherapy arm | Log-logistic θ=0.024470;κ=1.995940 |  |  |  |
| **HR for sintilimab+chemotherapy vs chemotherapy** | | | | |
| Total patients (OS) | 0.78 | 0.57-1.08 | Lognormal | The ORIENT-31 trial |
| Total patients (PFS) | 0.72 | 0.55-0.94 |  |  |
| Age<65 subgroup (PFS) | 0.72 | 0.53-0.97 |  |  |
| Age≥65 subgroup (PFS) | 0.69 | 0.42-1.14 |  |  |
| Male subgroup (PFS) | 0.80 | 0.54-1.20 |  |  |
| Female subgroup (PFS) | 0.65 | 0.46-0.91 |  |  |
| Baseline ECOG PS=0 subgroup (PFS) | 0.62 | 0.32-1.21 |  |  |
| Baseline ECOG PS=1 subgroup (PFS) | 0.72 | 0.54-0.95 |  |  |
| Baseline brain metastasis subgroup (PFS) | 0.84 | 0.54-1.29 |  |  |
| Non-baseline brain metastasis subgroup (PFS) | 0.64 | 0.46-0.88 |  |  |
| Non-Thr790Met mutations subgroup (PFS) | 0.59 | 0.43-0.81 |  |  |
| Thr790Met mutations subgroup (PFS) | 1.10 | 0.68-1.79 |  |  |
| Never smoked subgroup (PFS) | 0.66 | 0.48-0.90 |  |  |
| Current or former smoker subgroup (PFS) | 0.89 | 0.55-1.42 |  |  |
| Baseline liver metastases subgroup (PFS) | 0.72 | 0.32-1.61 |  |  |
| Non-Baseline liver metastases subgroup (PFS) | 0.69 | 0.53-0.91 |  |  |
| Previously received 1 line TKI treatment subgroup (PFS) | 0.57 | 0.42-0.78 |  |  |
| Previously received 2 line TKI treatment subgroup (PFS) | 1.30 | 0.80-2.10 |  |  |
| Exon 19 deletion subgroup (PFS) | 1.04 | 0.73-1.47 |  |  |
| Leu858Arg mutation subgroup (PFS) | 0.47 | 0.31-0.72 |  |  |
| Other EGFR mutation subgroup (PFS) | 0.36 | 0.12-1.09 |  |  |
| **HR for sintilimab+IBI305+chemotherapy vs chemotherapy** | | | | |
| Total patients (OS) | 0.79 | 0.57-1.09 | Lognormal | The ORIENT-31 trial |
| Total patients (PFS) | 0.51 | 0.39-0.67 |  |  |
| Age<65 subgroup (PFS) | 0.51 | 0.38-0.70 |  |  |
| Age≥65 subgroup (PFS) | 0.57 | 0.34-0.95 |  |  |
| Male subgroup (PFS) | 0.57 | 0.37-0.86 |  |  |
| Female subgroup (PFS) | 0.50 | 0.35-0.70 |  |  |
| Baseline ECOG PS=0 subgroup (PFS) | 0.30 | 0.17-0.55 |  |  |
| Baseline ECOG PS=1 subgroup (PFS) | 0.60 | 0.44-0.81 |  |  |
| Baseline brain metastasis subgroup (PFS) | 0.47 | 0.30-0.73 |  |  |
| Non-baseline brain metastasis subgroup (PFS) | 0.58 | 0.42-0.80 |  |  |
| Non-Thr790Met mutations subgroup (PFS) | 0.44 | 0.32-0.61 |  |  |
| Thr790Met mutations subgroup (PFS) | 0.87 | 0.52-1.46 |  |  |
| Never smoked subgroup (PFS) | 0.54 | 0.39-0.74 |  |  |
| Current or former smoker subgroup (PFS) | 0.50 | 0.31-0.81 |  |  |
| Baseline liver metastases subgroup (PFS) | 0.57 | 0.28-1.18 |  |  |
| Non-Baseline liver metastases subgroup (PFS) | 0.53 | 0.40-0.70 |  |  |
| Previously received 1 line TKI treatment subgroup (PFS) | 0.45 | 0.33-0.62 |  |  |
| Previously received 2 line TKI treatment subgroup (PFS) | 0.83 | 0.51-1.35 |  |  |
| Exon 19 deletion subgroup (PFS) | 0.81 | 0.57-1.15 |  |  |
| Leu858Arg mutation subgroup (PFS) | 0.37 | 0.24-0.57 |  |  |
| Other EGFR mutation subgroup (PFS) | 0.06 | 0.01-0.49 |  |  |

Abbreviations: OS, overall survival; PFS, progression-free survival; DSA, deterministic sensitivity analyses; PSA, probabilistic sensitivity analyses; HR, hazard ratio; ECOG, Eastern Cooperative Oncology Group; PS, performance status; TKI, tyrosine-kinase inhibitor; EGFR, epidermal growth factor receptor.
