# Supplementary material for: Sintilimab plus chemotherapy with or without bevacizumab biosimilar IBI305 in EGFR-mutated non-squamous NSCLC patients who progressed on EGFR TKI therapy: A China-based cost-effectiveness analysis
Source: PLoS One. 2024 Oct 18;19(10):e0312133. doi: 10.1371/journal.pone.0312133 (PMC11488704; doi:10.1371/journal.pone.0312133)
Supplement: S4 Table — (DOCX) [file pone.0312133.s004.docx]

**S4 Table. Derivation of AEs-related management costs**

| **AEs** | **Medication** | | **Examination** | | **Hospitalization** | | **Cost per event ($)** | **Reference** |
| --- | --- | --- | --- | --- | --- | --- | --- | --- |
|  | **Drugs** | **Cost^a^ ($)** | **Items** | **Cost^a^ ($)** | **Duration** | **Cost^a^ ($)** |  |  |
| Decreased neutrophil count decreased | Leukopoietin | 32.97 | Routine blood test | 8.47 | 7.00 | 52.04 | 93.49 | Expert consensus |
| Anaemia | Erythropoietin | 3.69 | Routine blood test | 8.47 | 5.00 | 37.17 | 49.34 | Expert consensus |
| Decreased white blood cell count | Leukopoietin | 32.97 | Routine blood test | 8.47 | 7.00 | 52.04 | 93.49 | Expert consensus |
| Nausea | Antiemetic | 13.14 | Electrolyte examination | 4.31 | 5.00 | 37.17 | 54.62 | Expert consensus |
| Decreased appetite | lutin | 1.16 | Electrolyte examination | 4.31 | 5.00 | 37.17 | 42.64 | Expert consensus |
| Asthenia | Blood tonics | 49.06 | Routine blood test | 5.65 | 7.00 | 52.04 | 106.75 | Local oncologists |
| Increased AST | Hepatinica | 11.42 | Liver function tests; abdominal CT | 108.83 | 5.00 | 37.17 | 157.42 | Expert consensus |
| Increased ALT | Hepatinica | 11.42 | Liver function tests; abdominal CT | 108.83 | 5.00 | 37.17 | 157.42 | Expert consensus |
| Vomiting | Antiemetic | 13.14 | Electrolyte examination | 4.31 | 5.00 | 37.17 | 54.62 | Expert consensus |
| Decreased platelet count | Interleukin | 205.62 | Routine blood test | 8.47 | 7.00 | 52.04 | 266.13 | Expert consensus |
| Hypertension | Depressor | 4.37 | Electrocardiogram;Echocardiography;Myocardial enzymes | 72.40 | 7.00 | 52.04 | 128.81 | Expert consensus |
| Increased γ-glutamyltransferase | Hepatinica | 11.42 | Liver function tests; abdominal CT | 108.83 | 5.00 | 37.17 | 157.42 | Expert consensus |
| Proteinuria | Glucocorticoid | 35.92 | Proteinuria assay | 1.49 | 7.00 | 52.04 | 89.44 | Local oncologists |
| Decreased lymphocyte count^b^ | / | / | / | / | / | / | 0.00 | Local oncologists |
| Hyperthyroidism | Antithyroid drugs | 4.58 | Thyroid function test | 29.73 | 7.00 | 52.04 | 86.35 | Local oncologists |
| Increased blood pressure | Depressor | 4.37 | Electrocardiogram;Echocardiography;Myocardial enzymes | 72.40 | 7.00 | 52.04 | 128.81 | Expert consensus |
| Rash | Antiallergic drug | 4.94 | Routine blood test | 5.65 | 5.00 | 37.17 | 47.76 | Expert consensus |
| Hypokalemia | Electrolyte | 0.18 | Electrolyte examination | 6.47 | 5.00 | 37.17 | 43.81 | Expert consensus |
| Pneumonitis | Glucocorticoid；Antibiotic | 56.14 | Pulmonary CT | 115.97 | 10.00 | 74.34 | 246.44 | Expert consensus |
| Diarrhoea | Antidiarrheal | 0.62 | Electrolyte examination | 4.31 | 5.00 | 37.17 | 42.10 | Expert consensus |
| Myelosuppression | G-CSF | 76.93 | Routine blood test | 8.47 | 7.00 | 52.04 | 137.44 | Local oncologists |
| Pneumonia | Glucocorticoid；Antibiotic | 56.14 | Pulmonary CT | 115.97 | 10.00 | 74.34 | 246.44 | Expert consensus |
| Interstitial lung disease | Glucocorticoid；Antibiotic | 56.14 | Pulmonary CT | 115.97 | 10.00 | 74.34 | 246.44 | Expert consensus |
| Increased blood triglycerides | Antihyperlipidemic | 0.69 | Lipid Profile | 16.21 | 7.00 | 52.04 | 68.93 | Expert consensus^7^ |
| Decreased granulocyte count | Leukopoietin | 32.97 | Routine blood test | 8.47 | 7.00 | 52.04 | 93.49 | Expert consensus |
| Electrolyte imbalance | Electrolyte | 75.00 | Electrolyte examination | 6.47 | 7.00 | 52.04 | 133.50 | Expert consensus |

Abbreviations: AEs, adverse events; ALT, alanine aminotransferase; AST, aspartate aminotransferase; CT, Computed Tomography; G-CSF, granulocyte colony-stimulating factor. ^a^These costs were estimated using data from local comprehensive hospitals.

^b^Decreased lymphocyte count does not require additional treatment, according to local oncologists’ opinion.
