# Supplementary material for: Sintilimab plus chemotherapy with or without bevacizumab biosimilar IBI305 in EGFR-mutated non-squamous NSCLC patients who progressed on EGFR TKI therapy: A China-based cost-effectiveness analysis
Source: PLoS One. 2024 Oct 18;19(10):e0312133. doi: 10.1371/journal.pone.0312133 (PMC11488704; doi:10.1371/journal.pone.0312133)
Supplement: S5 Table — (DOCX) [file pone.0312133.s005.docx]

**S5 Table. Derivation of AEs-Related Utility Decrements**

| **AEs** | **Disutilities** | **Source** | **Duration (Days)** | **Source** | **Disutilities Decrement** |
| --- | --- | --- | --- | --- | --- |
| Decreased neutrophil count decreased | 0.08973 | ICER | 16.80 | Delanoy N, et al | 0.00413 |
| Anaemia | 0.08973 |  | 42.00 | Delanoy N, et al | 0.01033 |
| Decreased white blood cell count | 0.08973 |  | 16.80 | Delanoy N, et al | 0.00413 |
| Nausea | 0.04802 |  | 14.00 | Borghaei H, et al | 0.00184 |
| Decreased appetite | 0.07346 |  | 14.00 | Borghaei H, et al | 0.00282 |
| Asthenia | 0.07346 |  | 5.00 | Msaouel P, et al | 0.00101 |
| Increased AST | 0.04680 |  | 56.70 | Borghaei H, et al | 0.00727 |
| Increased ALT | 0.04680 |  | 56.70 | Borghaei H, et al | 0.00727 |
| Vomiting | 0.04802 |  | 14.00 | Borghaei H, et al | 0.00184 |
| Decreased platelet count | 0.08973 |  | 30.80 | Delanoy N, et al | 0.00757 |
| Hypertension | 0.08973 |  | 28.00 | Local oncologists | 0.00688 |
| Increased γ-glutamyltransferase | 0.04680 |  | 56.70 | Borghaei H, et al | 0.00727 |
| Proteinuria | 0.04680 |  | 55.30 | Brahmer J, et al | 0.00709 |
| Decreased lymphocyte count | 0.08973 |  | 16.80 | Delanoy N, et al | 0.00413 |
| Hyperthyroidism | 0.04680 |  | 63.00 | Qiang Y | 0.00808 |
| Increased blood pressure | 0.08973 |  | 28.00 | Local oncologists | 0.00688 |
| Rash | 0.03248 |  | 56.70 | Borghaei H, et al | 0.00505 |
| Hypokalemia | 0.08973 |  | 55.30 | Brahmer J, et al | 0.01359 |
| Pneumonitis | 0.07346 |  | 41.30 | Borghaei H, et al | 0.00831 |
| Diarrhoea | 0.04680 |  | 14.00 | Borghaei H, et al | 0.00180 |
| Myelosuppression | 0.08973 |  | 56.00 | Delanoy N, et al | 0.01377 |
| Pneumonia | 0.07346 |  | 41.30 | Borghaei H, et al | 0.00831 |
| Interstitial lung disease | 0.04680 |  | 41.30 | Borghaei H, et al | 0.00530 |
| Increased blood triglycerides | 0.04680 |  | 56.70 | Borghaei H, et al | 0.00727 |
| Decreased granulocyte count | 0.08973 |  | 16.80 | Delanoy N, et al | 0.00413 |
| Electrolyte imbalance | 0.08973 |  | 55.30 | Brahmer J, et al | 0.01359 |

Abbreviations: AEs, adverse events; ALT, alanine aminotransferase; AST, aspartate aminotransferase; ICER, Institute for clinical and economic review.
