# Supplementary material for: Sintilimab plus chemotherapy with or without bevacizumab biosimilar IBI305 in EGFR-mutated non-squamous NSCLC patients who progressed on EGFR TKI therapy: A China-based cost-effectiveness analysis
Source: PLoS One. 2024 Oct 18;19(10):e0312133. doi: 10.1371/journal.pone.0312133 (PMC11488704; doi:10.1371/journal.pone.0312133)
Supplement: S6 Table — (DOCX) [file pone.0312133.s006.docx]

**S6 Table. The calculation of grades 3/4 AEs-induced costs and utilities for each treatment arm**

| **AEs** | **Proportion (%)** | | | **Cost per event($)** | **Disutility** |
| --- | --- | --- | --- | --- | --- |
|  | **Chemotherapy** | **Sintilimab+**  **Chemotherapy** | **Sintilimab+IBI305+**  **Chemotherapy** |  |  |
| Decreased neutrophil count decreased | 19% | 18% | 20% | 93.49 | 0.00413 |
| Anaemia | 12% | 8% | 13% | 49.34 | 0.01033 |
| Decreased white blood cell count | 9% | 9% | 11% | 93.49 | 0.00413 |
| Nausea | 1% | 3% | 3% | 54.62 | 0.00184 |
| Decreased appetite | 1% | 0% | 4% | 42.64 | 0.00282 |
| Asthenia | 2% | 1% | 3% | 106.75 | 0.00101 |
| Increased AST | 0% | 0% | 1% | 157.42 | 0.00727 |
| Increased ALT | 2% | 0% | 1% | 157.42 | 0.00727 |
| Vomiting | 1% | 1% | 3% | 54.62 | 0.00184 |
| Decreased platelet count | 5% | 6% | 8% | 266.13 | 0.00757 |
| Hypertension | 3% | 3% | 13% | 128.81 | 0.00688 |
| Increased γ-glutamyltransferase | 3% | 1% | 3% | 157.42 | 0.00727 |
| Proteinuria | 0% | 0% | 1% | 89.44 | 0.00709 |
| Decreased lymphocyte count | 1% | 1% | 3% | 0.00 | 0.00413 |
| Hyperthyroidism | 0% | 0% | 1% | 86.35 | 0.00808 |
| Increased blood pressure | 1% | 0% | 3% | 128.81 | 0.00688 |
| Rash | 1% | 1% | 0% | 47.76 | 0.00505 |
| Hypokalemia | 1% | 1% | 2% | 43.81 | 0.01359 |
| Pneumonitis | 0% | 2% | 1% | 246.44 | 0.00831 |
| Diarrhoea | 1% | 1% | 0% | 42.10 | 0.00180 |
| Myelosuppression | 1% | 1% | 3% | 137.44 | 0.01377 |
| Pneumonia | 1% | 1% | 2% | 246.44 | 0.00831 |
| Interstitial lung disease | 0% | 2% | 0% | 246.44 | 0.00530 |
| Increased blood triglycerides | 1% | 0% | 0% | 68.93 | 0.00727 |
| Decreased granulocyte count | 1% | 1% | 0% | 93.49 | 0.00413 |
| Electrolyte imbalance | 1% | 0% | 0% | 133.50 | 0.01359 |
| Estimated AEs costs and disutilities | | | |  |  |
| AEs cost for chemotherapy, $ | | | | 70.21 |  |
| AEs cost for sintilimab+chemotherapy, $ | | | | 69.81 |  |
| AEs cost for sintilimab+IBI305+chemotherapy, $ | | | | 107.50 |  |
| AEs disutility for chemotherapy, $ | | | | | 0.00421 |
| AEs disutility for sintilimab+chemotherapy, $ | | | | | 0.00354 |
| AEs disutility for sintilimab+IBI305+chemotherapy, $ | | | | | 0.00616 |

Abbreviations: AEs, adverse events; ALT, alanine aminotransferase; AST, aspartate aminotransferase.
