# Supplementary material for: Sintilimab plus chemotherapy with or without bevacizumab biosimilar IBI305 in EGFR-mutated non-squamous NSCLC patients who progressed on EGFR TKI therapy: A China-based cost-effectiveness analysis
Source: PLoS One. 2024 Oct 18;19(10):e0312133. doi: 10.1371/journal.pone.0312133 (PMC11488704; doi:10.1371/journal.pone.0312133)
Supplement: S7 Table — (DOCX) [file pone.0312133.s007.docx]

**S7 Table. Model inputs regarding costs and health state utilities** **estimation**

| **Model inputs** | **Baseline Value** | **Range for DSA** | **Distribution for PSA** | **Source** |
| --- | --- | --- | --- | --- |
| **Costs (US$)** | | | | |
| Sintilimab per 200mg | 321.14 | 240.85-401.42 | Gamma | National Health Industry Data Platform |
| IBI305 per 15mg/kg | 24.89 | 18.67-31.11 | Gamma |  |
| Pemetrexed per 500mg/m^2^ | 269.58 | 202.18-336.97 | Gamma |  |
| Cisplatin per 75mg/m^2^ | 8.89 | 6.67-11.11 | Gamma |  |
| Sub-anticancer therapy per cycle | 115.93 | 86.94-144.91 | Gamma |  |
| Routine follow-up per cycle | 55.60 | 41.70-69.50 | Gamma | Luo X, et al |
| BSC per cycle | 337.50 | 253.13-421.88 | Gamma |  |
| Palliative care per cycle | 2627.80 | 1970.85-3284.75 | Gamma |  |
| AEs cost in the chemotherapy arm | 70.21 | 52.66-87.77 | Gamma | Supplementary Table S6 |
| AEs cost in the sintilimab+chemotherapy arm | 69.81 | 52.36-87.27 | Gamma |  |
| AEs cost in the sintilimab+IBI305+chemotherapy arm | 107.50 | 80.63-134.28 | Gamma |  |
| **Health state utilities** | | | | |
| SD health state of the chemotherapy arm | 0.75175 | 0.56381-0.93969 | Beta | Shen Y, et al |
| SD health state of the sintilimab+chemotherapy arm | 0.75154 | 0.56366-0.93943 | Beta |  |
| SD health state of the sintilimab+IBI305+chemotherapy arm | 0.74949 | 0.56212-0.93686 | Beta |  |
| PD health state | 0.70300 | 0.52725-0.87875 | Beta |  |
| AEs disutility in the chemotherapy arm | 0.00421 | 0.00316-0.00527 | Beta | Supplementary Table S6 |
| AEs disutility in the sintilimab+chemotherapy arm | 0.00354 | 0.00265-0.00442 | Beta |  |
| AEs disutility in the sintilimab+IBI305+chemotherapy arm | 0.00616 | 0.00462-0.00770 | Beta |  |
| **Other** |  |  |  |  |
| Discount rate | 0.05 | 0.00-0.08 | Fixed | China Guidelines for Pharmacoeconomic Evaluations |
| Body surface area(m^2^) | 1.72 | 1.29-2.15 | Normal | Luo X, et al |
| Mean weight (kg) | 63.35 | 47.51-79.18 | Normal |  |
| Male weight (kg) | 69.60 | 52.20-87.00 | Normal | National Health Commission of the People's Republic of China |
| Female weight (kg) | 59.00 | 44.25-73.75 | Normal |  |

Abbreviations: DSA, deterministic sensitivity analyses; PSA, probabilistic sensitivity analyses; BSC, best supportive care; AEs, advent events; SD, stable disease; PD, progressed disease.
