# Supplementary material for: Sintilimab plus chemotherapy with or without bevacizumab biosimilar IBI305 in EGFR-mutated non-squamous NSCLC patients who progressed on EGFR TKI therapy: A China-based cost-effectiveness analysis
Source: PLoS One. 2024 Oct 18;19(10):e0312133. doi: 10.1371/journal.pone.0312133 (PMC11488704; doi:10.1371/journal.pone.0312133)
Supplement: S8 Table — (DOCX) [file pone.0312133.s008.docx]

**S8 Table. Subgroup-level analysis results**

| **Subgroup** | **Strategy** | **Costs** | **QALYs** | **Incremental costs (vs chemotherapy)** | **Incremental QALYs**  **(vs chemotherapy)** | **ICER ($/QALY)** |
| --- | --- | --- | --- | --- | --- | --- |
| Age <65 | Chemotherapy | 21,322.48 | 1.64024 | NA | NA | NA |
|  | Sintilimab+chemotherapy | 27,287.22 | 2.02936 | 5,964.73 | 0.38911 | 15,329.11 |
|  | Sintilimab+IBI305+chemotherapy | 42,036.20 | 2.02912 | 20,713.72 | 0.38887 | 53,266.32 |
| Age ≥65 | Chemotherapy | 21,322.48 | 1.64024 | NA | NA | NA |
|  | Sintilimab+chemotherapy | 27,279.17 | 2.03166 | 5,956.69 | 0.39142 | 15,218.27 |
|  | Sintilimab+IBI305+chemotherapy | 42,064.78 | 2.02117 | 20,742.30 | 0.38092 | 54,452.99 |
| Male | Chemotherapy | 21,322.48 | 1.64024 | NA | NA | NA |
|  | Sintilimab+chemotherapy | 27,305.12 | 2.02425 | 5,982.64 | 0.38401 | 15,579.54 |
|  | Sintilimab+IBI305+chemotherapy | 43,464.85 | 2.02117 | 22,142.36 | 0.38092 | 58,128.45 |
| Female | Chemotherapy | 21,322.48 | 1.64024 | NA | NA | NA |
|  | Sintilimab+chemotherapy | 27,267.06 | 2.03515 | 5,944.57 | 0.39490 | 15,053.33 |
|  | Sintilimab+IBI305+chemotherapy | 41,056.52 | 2.03058 | 19,734.04 | 0.39033 | 50,557.20 |
| Baseline ECOG PS=0 | Chemotherapy | 21,322.48 | 1.64024 | NA | NA | NA |
|  | Sintilimab+chemotherapy | 27,256.76 | 2.03811 | 5,934.28 | 0.39787 | 14,915.12 |
|  | Sintilimab+IBI305+chemotherapy | 41,901.04 | 2.06654 | 20,578.56 | 0.42630 | 48,272.90 |
| Baseline ECOG PS=1 | Chemotherapy | 21,322.48 | 1.64024 | NA | NA | NA |
|  | Sintilimab+chemotherapy | 27,287.22 | 2.02936 | 5,964.73 | 0.38911 | 15,329.11 |
|  | Sintilimab+IBI305+chemotherapy | 42,077.25 | 2.01773 | 20,754.77 | 0.37749 | 54,981.07 |
| Baseline brain metastasis | Chemotherapy | 21,322.48 | 1.64024 | NA | NA | NA |
|  | Sintilimab+chemotherapy | 27,312.50 | 2.02216 | 5,990.02 | 0.38191 | 15,684.20 |
|  | Sintilimab+IBI305+chemotherapy | 42,014.43 | 2.03518 | 20,691.95 | 0.39494 | 52,393.15 |
| Non-baseline brain metastasis | Chemotherapy | 21,322.48 | 1.64024 | NA | NA | NA |
|  | Sintilimab+chemotherapy | 27,263.75 | 2.03610 | 5,941.26 | 0.39585 | 15,008.72 |
|  | Sintilimab+IBI305+chemotherapy | 42,069.08 | 2.01998 | 20,746.59 | 0.37974 | 54,634.39 |
| Non-Thr790Met mutations | Chemotherapy | 21,322.48 | 1.64024 | NA | NA | NA |
|  | Sintilimab+chemotherapy | 27,245.27 | 2.04144 | 5,922.78 | 0.40119 | 14,762.93 |
|  | Sintilimab+IBI305+chemotherapy | 41,996.71 | 2.04011 | 20,674.22 | 0.39987 | 51,702.59 |
| Thr790Met mutations | Chemotherapy | 21,322.48 | 1.64024 | NA | NA | NA |
|  | Sintilimab+chemotherapy | 27,344.43 | 2.01321 | 6,021.95 | 0.37296 | 16,146.29 |
|  | Sintilimab+IBI305+chemotherapy | 42,146.18 | 1.99902 | 20,823.69 | 0.35878 | 58,040.22 |
| Never smoked | Chemotherapy | 21,322.48 | 1.64024 | NA | NA | NA |
|  | Sintilimab+chemotherapy | 27,270.25 | 2.03423 | 5,947.76 | 0.39398 | 15,096.54 |
|  | Sintilimab+IBI305+chemotherapy | 42,051.10 | 2.02496 | 20,728.62 | 0.38472 | 53,879.68 |
| Current or former smoker | Chemotherapy | 21,322.48 | 1.64024 | NA | NA | NA |
|  | Sintilimab+chemotherapy | 27,320.55 | 2.01989 | 5,998.07 | 0.37964 | 15,799.31 |
|  | Sintilimab+IBI305+chemotherapy | 42,030.96 | 2.03058 | 20,708.48 | 0.39033 | 53,053.66 |
| Baseline liver metastases | Chemotherapy | 21,322.48 | 1.64024 | NA | NA | NA |
|  | Sintilimab+chemotherapy | 27,287.22 | 2.02936 | 5,964.73 | 0.38911 | 15,329.11 |
|  | Sintilimab+IBI305+chemotherapy | 42,064.78 | 2.02117 | 20,742.30 | 0.38092 | 54,452.99 |
| Non-baseline liver metastases | Chemotherapy | 21,322.48 | 1.64024 | NA | NA | NA |
|  | Sintilimab+chemotherapy | 27,279.17 | 2.03166 | 5,956.69 | 0.39142 | 15,218.27 |
|  | Sintilimab+IBI305+chemotherapy | 42,046.27 | 2.02631 | 20,723.79 | 0.38607 | 53,679.48 |
| Previously received 1 line TKI treatment | Chemotherapy | 21,322.48 | 1.64024 | NA | NA | NA |
|  | Sintilimab+chemotherapy | 27,236.86 | 2.04387 | 5,914.38 | 0.40363 | 14,653.01 |
|  | Sintilimab+IBI305+chemotherapy | 42,002.75 | 2.03843 | 20,680.26 | 0.39819 | 51,935.76 |
| Previously received 2 lines TKI treatment | Chemotherapy | 21,322.48 | 1.64024 | NA | NA | NA |
|  | Sintilimab+chemotherapy | 27,358.24 | 2.00938 | 6,035.75 | 0.36914 | 16,351.05 |
|  | Sintilimab+IBI305+chemotherapy | 42,139.37 | 2.00085 | 20,816.89 | 0.36060 | 57,727.90 |
| Exon 19 deletion | Chemotherapy | 21,322.48 | 1.64024 | NA | NA | NA |
|  | Sintilimab+chemotherapy | 27,338.89 | 2.01475 | 6,016.40 | 0.37450 | 16,064.95 |
|  | Sintilimab+IBI305+chemotherapy | 42,135.65 | 2.00185 | 20,813.16 | 0.36160 | 57,557.77 |
| Leu858Arg mutation | Chemotherapy | 21,322.48 | 1.64024 | NA | NA | NA |
|  | Sintilimab+chemotherapy | 27,186.63 | 2.05862 | 5,864.15 | 0.41837 | 14,016.54 |
|  | Sintilimab+IBI305+chemotherapy | 41,951.08 | 2.05276 | 20,628.60 | 0.41251 | 50,007.09 |
| other EGFR mutation | Chemotherapy | 21,322.48 | 1.64024 | NA | NA | NA |
|  | Sintilimab+chemotherapy | 27,116.25 | 2.07922 | 5,793.77 | 0.43897 | 13,198.49 |
|  | Sintilimab+IBI305+chemotherapy | 41,783.35 | 2.09864 | 20,460.87 | 0.45840 | 44,635.66 |

Abbreviations: QALYs, quality-adjusted life-years; ICERs, incremental cost-effectiveness ratios; NA, not applicable; ECOG, Eastern Cooperative Oncology Group; PS, performance status; TKI, tyrosine-kinase inhibitor; EGFR, epidermal growth factor receptor.
