# Supplementary figures and images for: Sintilimab plus chemotherapy with or without bevacizumab biosimilar IBI305 in EGFR-mutated non-squamous NSCLC patients who progressed on EGFR TKI therapy: A China-based cost-effectiveness analysis
Source: PLoS One. 2024 Oct 18;19(10):e0312133. doi: 10.1371/journal.pone.0312133 (PMC11488704; doi:10.1371/journal.pone.0312133)

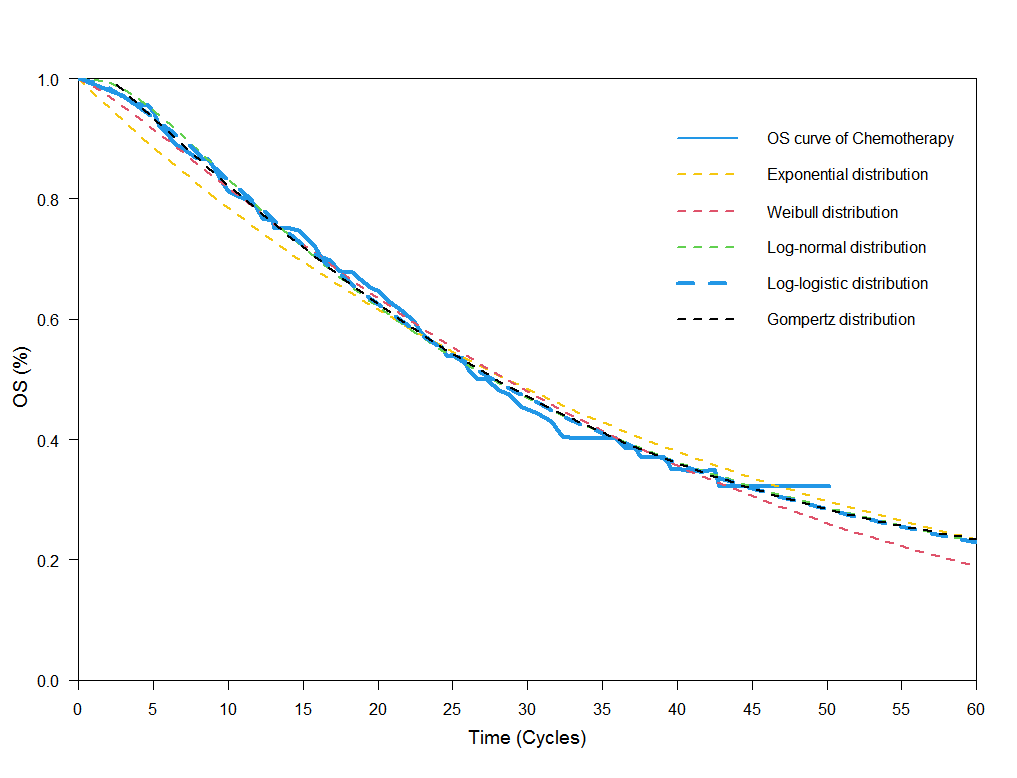

Supplement: S1 Fig — KM, Kaplan-Meier; OS, overall survival. (TIF) [file pone.0312133.s009.tif]

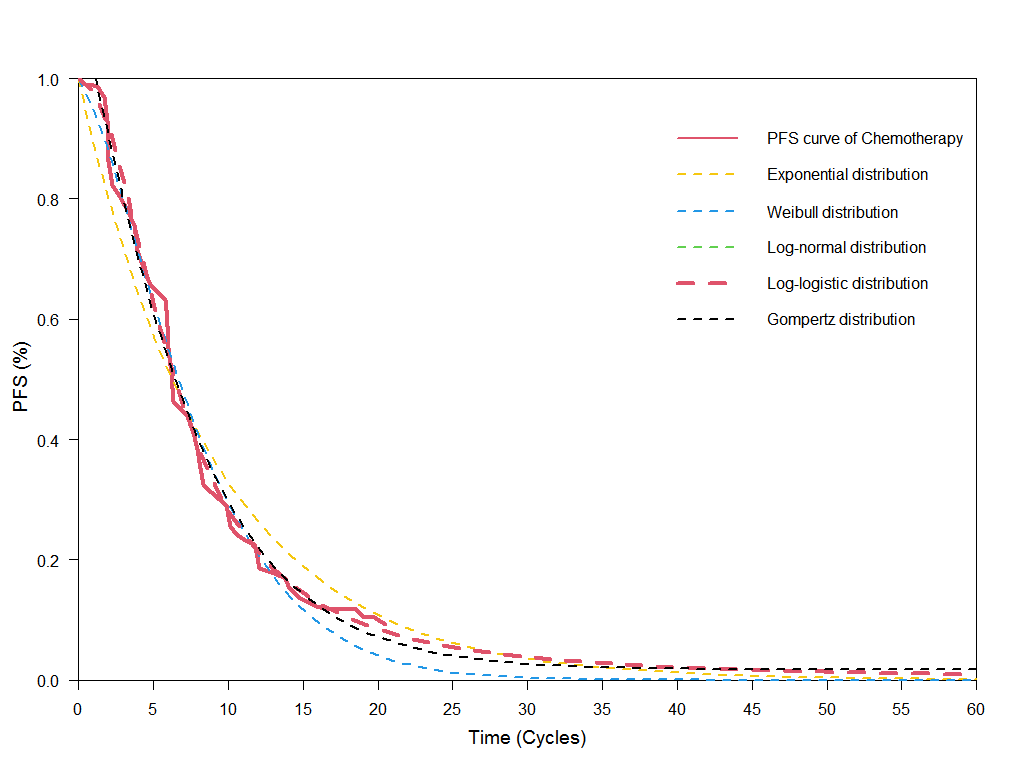

Supplement: S2 Fig — KM, Kaplan-Meier; PFS, progression-free survival. (TIF) [file pone.0312133.s010.tif]
